# Unveiling Multiquantum Excitonic Correlations in Push–Pull Polymer Semiconductors

**DOI:** 10.1021/acs.jpclett.4c00065

**Published:** 2024-03-28

**Authors:** Yulong Zheng, Esteban Rojas-Gatjens, Myeongyeon Lee, Elsa Reichmanis, Carlos Silva-Acuña

**Affiliations:** † School of Chemistry and Biochemistry, Georgia Institute of Technology, 901 Atlantic Drive, Atlanta, Georgia 30332, United States; ‡ Department of Chemical & Biomolecular Engineering, Lehigh University, 124 E. Morton Street, Bethlehem, Pennsylvania 18015, United States; § Institut Courtois & Département de physique, Université de Montréal, 1375 Avenue Thérèse-Lavoie-Roux, Montréal, Québec H2V 0B3, Canada

## Abstract

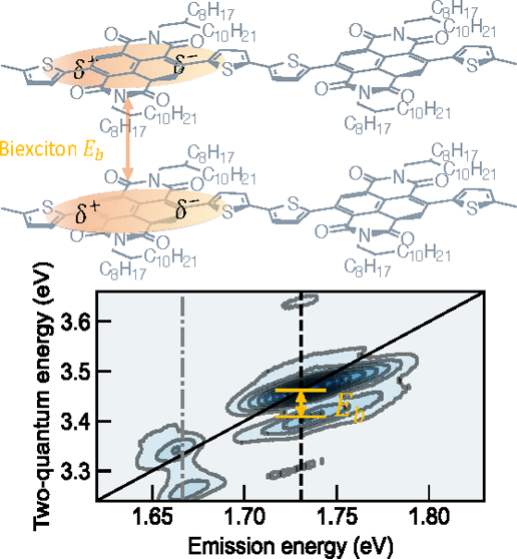

Bound and unbound Frenkel-exciton pairs are essential transient precursors for a variety of photophysical and biochemical processes. In this work, we identify bound and unbound Frenkel-exciton complexes in an electron push–pull polymer semiconductor using coherent two-dimensional spectroscopy. We find that the dominant A_0–1_ peak of the absorption vibronic progression is accompanied by a subpeak, each dressed by distinct vibrational modes. By considering the Liouville pathways within a two-exciton model, the imbalanced cross-peaks in one-quantum rephasing and nonrephasing spectra can be accounted for by the presence of pure biexcitons. The two-quantum nonrephasing spectra provide direct evidence for unbound exciton pairs and biexcitons with dominantly attractive force. In addition, the spectral features of unbound exciton pairs show mixed absorptive and dispersive character, implying many-body interactions within the correlated Frenkel-exciton pairs. Our work offers novel perspectives on the Frenkel-exciton complexes in semiconductor polymers.

Frenkel excitons are a collective of local excitations coupled through resonant Coulomb interactions within chromophores.^1^ Despite the fact that the extent of the delocalization can theoretically span the entirety of the aggregate structure, no realistic molecular aggregates are disorder-free, especially in conjugated polymers, where both static disorder (e.g., conformational disorder from site to site) and environmental fluctuations (e.g., low-frequency torsional modes) significantly constrain the effective delocalization length.^2−5^ As a consequence, at sufficiently high excitation densities, multiple excitons can coexist in close proximity, leading to distinguishable exciton–exciton interactions and correlations.^6^ Electron push–pull polymers are known to form disordered polymeric aggregates,^7,8^ which could host two-dimensional hybrid HJ excitons.^2,3,9−11^ In this work, we show direct evidence of two distinct excitons dressed by different vibrational modes, each with its own vibronic progression. Furthermore, we demonstrate the presence of Frenkel biexcitons and correlated exciton pairs revealed in one-quantum (1Q) and two-quantum (2Q) two-dimensional coherent spectra (2DCS). By tracing the Liouville pathways qualitatively on a two-exciton basis, we show that the spectral overlap between the biexcitons and the dominant feature of single excitons gives rise to asymmetric cross-peaks in the 1Q spectra.

Under a two-level molecular picture, Frenkel exciton–exciton interactions (EEI) can be categorized into two types: the first type, termed kinematic exciton–exciton interactions, originates from the hard-core-like scattering between Frenkel excitons due to the Pauli exclusion principle.^12^ Naturally, the kinematic interaction gives rise to an effective repulsive two-exction state, which is observed as a blue-shifted positive absorption feature (in differential absorption) relative to the ground-state bleach for J-aggregates in pump–probe experiments.^13−15^ The second type is the dynamic exciton–exciton interaction originating from the differences in the permanent static dipoles of the ground and excited states.^16^ For the latter case, experimental reports on the existence of dynamic Frenkel biexcitons in molecular aggregates are fairly limited, with sporadic evidence provided by fluence-dependent intensity and spectral line shape analysis by transient absorption measurements.^17−19^ Recently, more direct evidence is presented through the use of multiquantum coherent spectroscopy.^20−23^ Dostál et al. ascribed the growing two-exciton features in a small-molecule aggregated system to exciton–exciton interactions through diffusion.^20^ Malý et al. probed exciton transport transitioning from wavelike to subdiffusive behavior through EEI in a conjugated copolymer with varying chain lengths.^21^ Gutiérrez-Meza et al. investigated the correlation between the biexcitonic binding energy and hybrid H and J aggregate characteristics in a liquid-crystalline-like conjugated polymer.^23,24^ Despite the incremental new discoveries of Frenkel-exciton properties, the biexciton resonances and many-body correlations (e.g., excitation-induced dephasing) in Frenkel-exciton systems are not well developed as in their Wannier–Mott counterparts.^25−32^ In addition, although the biexcitons are observed explicitly in the 2Q spectra, their contributions to the 1Q spectra are often neglected.

In this Letter, we address these issues by probing the conjugated electron push–pull polymer poly[*N*,*N*′-bis(2-octyldodecyl)naphthalene-1,4,5,8-bis(dicarboximide)-2,6-diyl]-*alt*-5,5′-(2,2′-bithiophene) (or N2200, and its associated chemical structure is shown in Figure 1a) by means of two-dimensional coherent spectroscopic measurements. The thin-film preparation of N2200 is described in the Supporting Information. Compared to conjugated homopolymers, the strong charge-transfer character in the electron push–pull polymers leads to a large permanent static dipole moment,^33^ which determines the strength of dynamic EEI.^6^ Another difference lies in the fact that the electronic transitions in conjugated copolymers are coupled to more vibrational modes, resulting in synergistic intermode effects, where the weakly coupled mode could borrow intensities from the strongly coupled vibrational mode.^34^ These, along with polymorphism and static disorder, lead to significantly congested spectral features in electron push–pull polymers.

**Figure 1:**
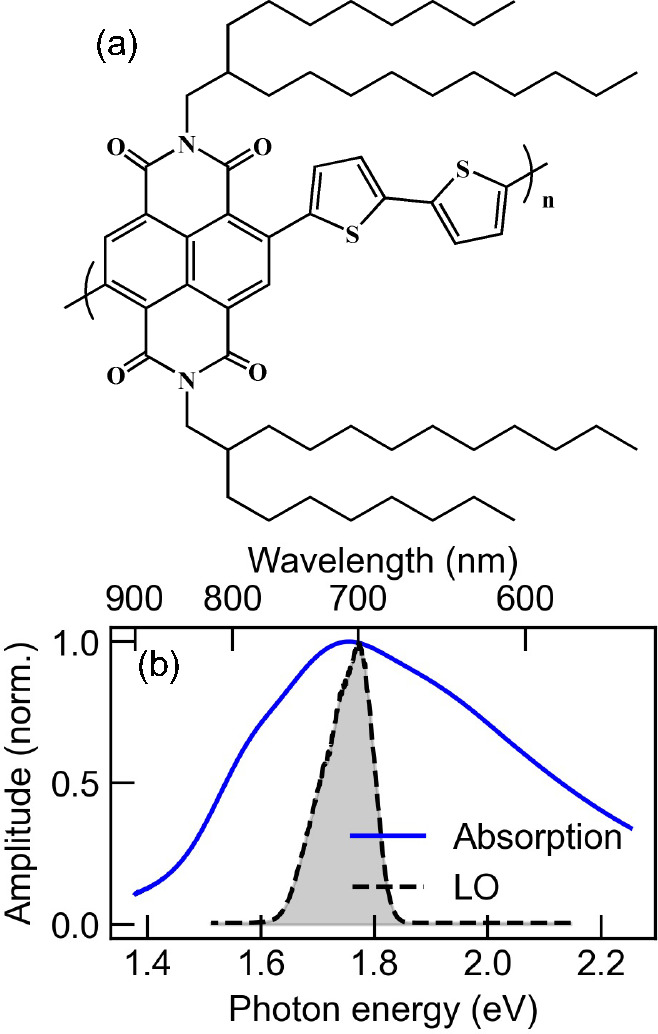
(a) Chemical structure of N2200. (b) Absorption spectra of the low-energy band of N2200 (blue solid line) and the femtosecond pulse spectrum of the local oscillator used in the 2DCS measurements reported in this paper (black dashed line shaded in gray).

Implementing 2DCS, we directly resolve (i) electronic correlations between different excited states and (ii) inhomogeneous and homogeneous broadening contributions into the optical line widths.^35^ We employ a coherent optical laser beam recombination technique (COLBERT) designed in the research group of Keith Nelson,^36,37^ which adopts a four-wave-mixing (FWM) signal acquisition scheme based on phase matching imposed by the incident beam geometry and on time ordering of the femtosecond pulse sequence. This spectroscopy generates a third-order macroscopic coherent polarization by resonantly interacting a pulse train of three sequential beams with an optically active material resonantly. The coherent emission propagates in the well-defined direction for one-quantum rephasing scheme with wavevector  and nonrephasing scheme with wavevector , where the difference lies on the relative pulse arrival within the two first phase-conjugate pulses. Eventually, the coherent signal is detected through spectral interferometry by an attenuated fourth beam (i.e., the local oscillator or LO). Fourier-transforming along the first and third time duration variable gives rise to correlated “absorption” and “emission” axes, where the different electronic transitions lie on the diagonal axis, but any correlations between the electronic states show up as cross-peaks.^38^ The experimental method is further explained in the Supporting Information. In this work, we performed a series of fluence-dependent measurements with the pulse fluence varied from 12.8 to 121 μJ/cm^2^ at an initial population waiting time of 20 fs (to avoid contamination from coherent artifacts at shorter delays). Here, we display a case measured at an intermediate fluence (Figure 2), with the rest shown in Figure S2, where we observed no drastic fluence dependence of the spectral line shape. We overlapped the pulse spectrum with the A_0–1_ vibronic transition (Figure 2a) in the N2200 thin film. The real, imaginary, and absolute part of the 1Q rephasing diagrams are shown in Figures 2b, 2c, and 2d, respectively. The real part is absorptive, while the imaginary part is dispersive *along* the diagonal axis, as expected in a transmission experiment. Interestingly, the absorptive feature in Figure 2b appears to be elongated along the diagonal axis with a slight tail that extends along the absorption energy axis, which is enhanced in the absolute diagram in Figure 2d. In addition, the cross-peaks in Figure 2b seem to be negative even though they are more attenuated compared to the dominant peaks. We will discuss their implications when considering all possible Liouville pathways later. As the dephasing rate determines the homogeneous line width, we also took the antidiagonal cuts of the absolute-valued spectra as shown in Figure S3. Despite the distinct pumping fluences, we observed no drastic differences between the antidiagonal line widths, as opposed to what was previously observed in inorganic and perovskite semiconductors, which was attributed to excitation-induced dephasing.^25,27,29^ Such a difference might be due to the strongly bound nature of Frenkel excitons in comparison to Wannier–Mott excitons, where Frenkel excitons are less susceptible to long-range Coulombic screening, at least in this type of push–pull conjugated polymer. In contrast to rephasing spectra, the real (Figure 2f) and imaginary part (Figure 2g) of the nonrephasing spectrum demonstrate absorptive and dispersive characteristic *across* the diagonal axis, respectively. A small side peak is observed above the dominant A_0–1_ peak, which might be due to the interstate coherence with A_0–2_ that is out of the spectral range. Because the phase twist is now perpendicular to the diagonal axis, the shoulder at 1.664 eV along the diagonal axis and cross-peak at (1.736, 1.664) eV emerge more clearly. The same character is also observed in the absolute-value diagram for nonrephasing in Figure 2h.

**Figure 2:**
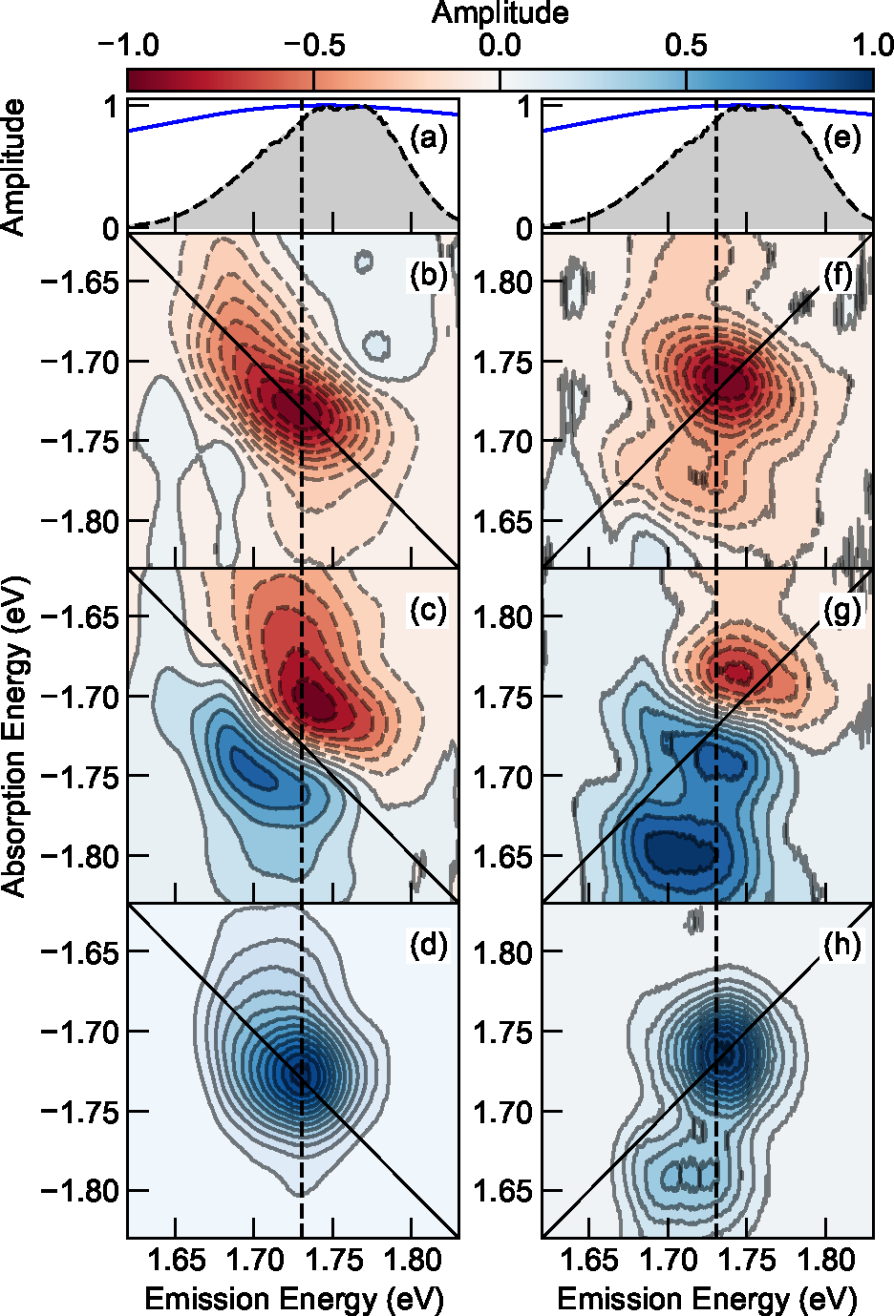
(a, e) Absorption spectra of A_0–1_ in blue solid curve with the pulse spectra shown in dashed black line shaded in gray. (b–d) Real, imaginary, and absolute spectrum of the rephasing diagram, measured with a fluence of 25.6 μJ/cm^2^. (f–h) Real, imaginary, and absolute spectrum of the nonrephasing diagram. All measurements are conducted with the samples positioned in a high-vacuum chamber at ambient temperature.

A common practice to eliminate the phase twist issue is to sum the real part of rephasing and nonrephasing diagram, in which the line width is purely absorptive.^39^ By doing so, the small shoulder becomes more prevalent besides the dominant A_0–1_ transition as shown in Figure 3a–d. It is worth noting that the peak amplitude is weighted by the product of the absorption spectrum and the pulse intensity. As the intensity of the beam is much more attenuated on the low-energy part, this feature should be much stronger than it appears. The cross-peak at (1.736, 1.656) eV indicates that the two excitons share a common ground state, which excludes the possibility of the side peak originating from a different polymer phase. In addition, we also want to highlight that the subpeak cannot be the tail of A_0–0_ as its energy difference from the A_0–1_ is less than 80 meV, greatly smaller than the energy of the dominant vinyl-stretching mode (170 meV), characteristic of various conjugated polymers.^40^ Another important feature is the asymmetric cross-peaks in the upper and lower quadrants of the 2D spectrum, also seen in the nonrephasing diagram. Such a signature was explained previously for a cancellation of the Liouville pathways for the interstate coherence and excited state absorption (of mixed biexciton states; see below).^41^ The cross-peak amplitude would then scale as the coupling strength between the two excitons in the weak-coupling limit. In the upper quadrant, the cross-peak shown in the red dashed square has a positive sign, as opposed to the dominant features. Because they overlap with the dominant feature, their real intensities might be underestimated.

**Figure 3:**
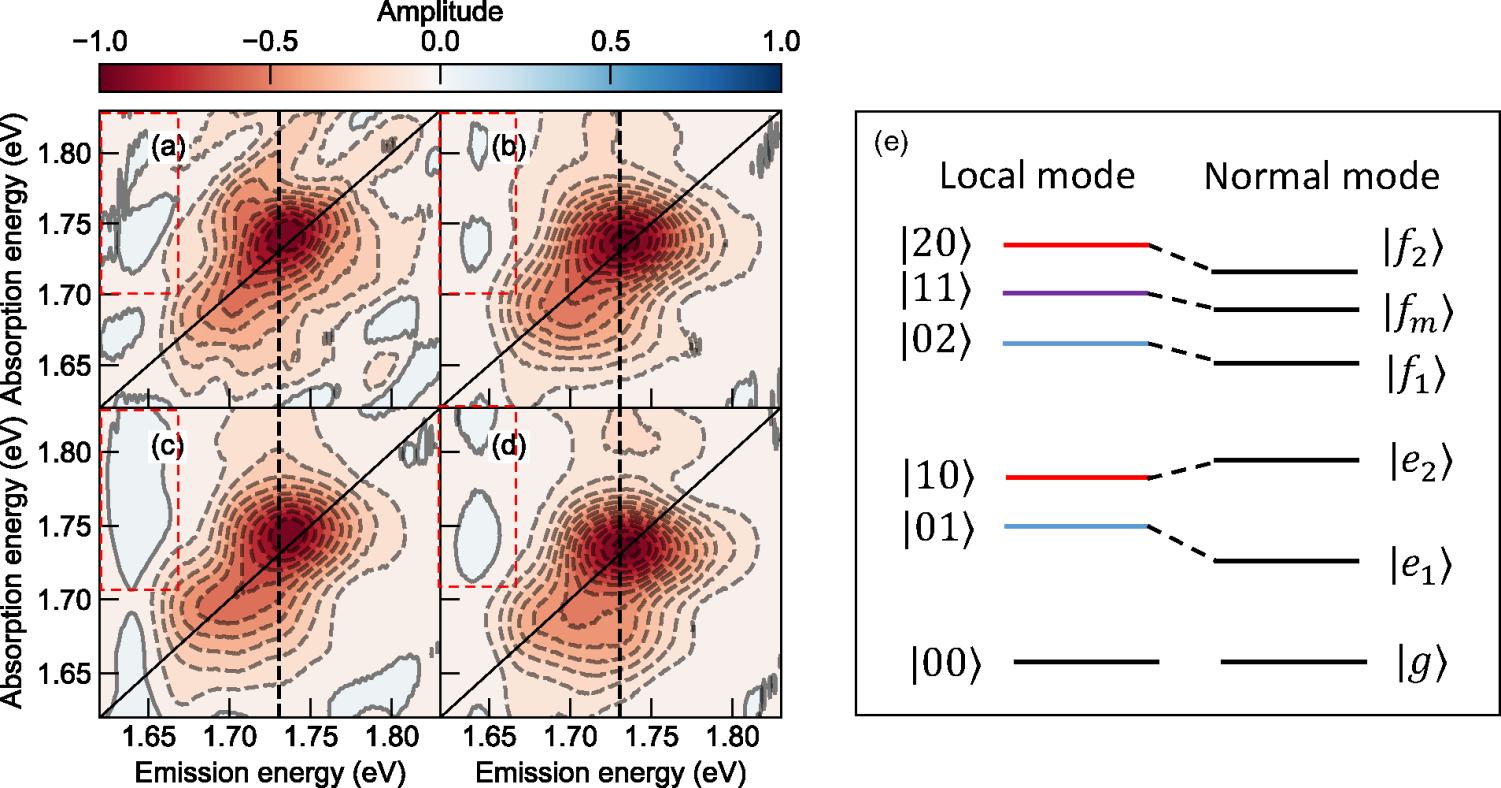
(a–d) 1Q total-correlation spectroscopy of 12.8, 25.6, 51.2, and 121 μJ/cm^2^, respectively. The black dashed lines indicate the position of the dominant A_0–1_ feature. The red dashed squares highlight positive features indicating the contribution from biexcitons. (e) Level scheme for both local and normal mode system of two heterogeneous vibronic excitons and their associated biexcitons. The relative energies between the normal modes depend upon the sign and magnitude of exciton–exciton coupling strengths.

By identifying the two one-exciton transitions, we can apply the level scheme of a pair of heterogeneous vibronic excitons with their associated biexciton states as shown in Figure 3e.^39^ |*n*_ν_1__*m*_ν_2__⟩ denotes a state that has *m* excitons, each coupled to the dominant vinyl-stretching vibrational mode, ν_2_, and *n* excitons coupled to the satellite vibrational mode, ν_1_. As the relative positions of |*n*⟩ and |*m*⟩ can encode the two vibration modes directly, we discard the subscription in the following discussion for simplicity. For FWM experiments, only a conserved two-exciton space needs to be considered. Specifically, we only take account of the pure biexciton states, |20⟩ and |02⟩, and mixed biexciton states, |11⟩. Transitions are ignored when multiple transition dipoles are required. For example, a direct transition, |10⟩ → |02⟩ is considered forbidden because it involves multiple photons in one step. The associated normal mode is given schematically in the right panel of Figure 3e. The exact energy shift depends on the magnitude and sign of the exciton–exciton coupling operators.

The Liouville pathways considering all allowed transitions are demonstrated in Figure 4. Despite the fact that the line widths in conjugated polymers are broadened, qualitative features can be observed immediately. The positive features that originate from transitions to the mixed biexciton (|*f*_*m*_⟩) and pure biexciton (|*f*_1_⟩, |*f*_2_⟩) states concentrate on the left regime to the diagonal axis, while the right side of the diagonal axis has an overlapped feature from the excited state absorption of the mixed biexciton and interstate coherence as mentioned earlier in both rephasing and nonrephasing spectra. The overall imbalanced 1Q total correlation spectra are indeed observed, as shown in Figure 3a–d. However, here we considered all three biexciton states, where the pure biexciton features also give rise to positive features even though they could have larger energy shift. Therefore, the overall imbalanced spectral features could be caused by (1) the overlap between the mixed biexciton absorption and interstate coherence on both sides of the diagonal axis with their amplitude determined by the EEI strength and their relative transition dipole moments, (2) pure biexciton absorption on the left regime, leaving the right regime in negative sign due to the interstate coherence solely, or (3) a combination of both contributions. To address this issue, we resort to the 2Q nonrephasing scheme in the phase-matching direction .^42^ In contrast to the 1Q, the first two pulses interacting with the material share the same phase. The sequential excitation could generate biexciton and unbound exciton pairs, which can be resolved in more detail along the two-quantum axis.^28,43^

**Figure 4:**
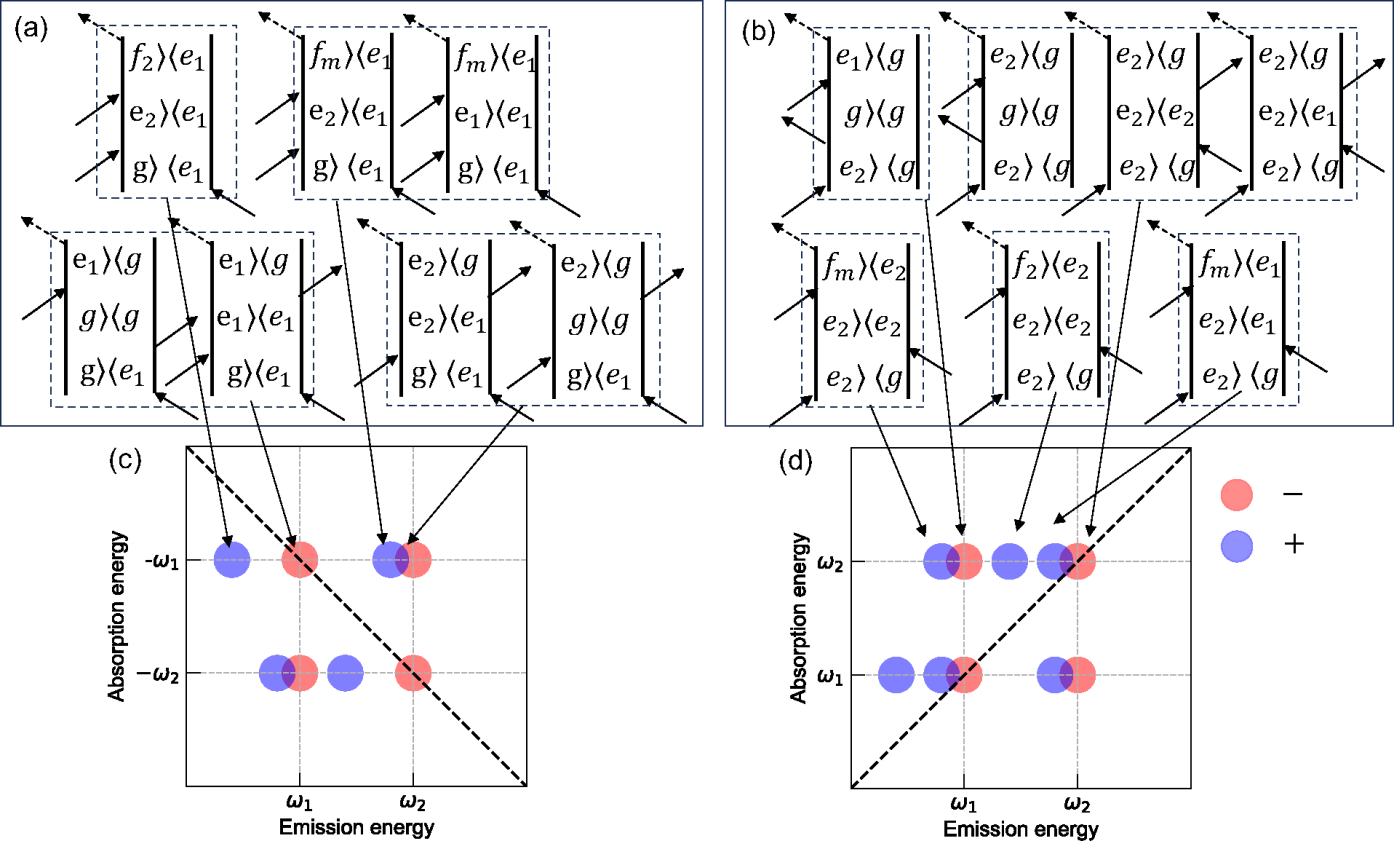
Liouville pathways for rephasing (a) and nonrephasing (b) diagrams. Schematic of purely absorptive 1Q spectra for rephasing (c) and nonrephasing (d) phase matching conditions. The negative and positive features are denoted in red and blue, respectively. Figure inspired originally by Figures 4.11 and 4.13 from ref (39).

To more easily compare the 2Q spectral features with their 1Q counterparts, the 1Q and 2Q nonrephasing measurements are presented in Figure 5 under the pumping fluence of each pulse being 121 μJ/cm^2^. The spectral features do not significantly depend on the fluences, although low-fluence measurements seem to present more artifacts as shown in Figure S4. A close match of the energies of the two heterogeneous vibronic excitons in 1Q and 2Q spectra can be found by the red and black dashed lines. Two prominent features can be observed and are explained in Figure 5d–f. First, the two dominant peaks, β and α, reside on the diagonal axis, each accompanied by a red-shifted side peak, β′ and α′, respectively, along the two-quantum axis. Therefore, the binding energies, experimentally determined as (*E*_2Q_ – 2*E*_1Q_), for |*f*_1_⟩ and |*f*_2_⟩ are estimated to be −76 and −64 meV as shown in Figure S6, respectively, where the negative sign indicates their attractive nature. The exciton binding energies are comparable because the two vibronic excitons have the same electronic origins, while the slight difference might originate from the perturbation of the two distinct vibrational modes. Interestingly, a blue-shifted shoulder around (1.736, 3.502) eV can also be observed extending out of α. Therefore, the repulsive binding energy can be estimated to be around 39 meV. One possible origin of such a positive feature could be the kinematic exciton–exciton scattering mentioned above. Second, unlike the real and imaginary part of the 1Q nonrephasing spectrum in Figures 5a and 5b, which show distinct absorptive and dispersive features, respectively, the real and imaginary parts of the 2Q nonrephasing spectrum show mixed features. Such features are previously observed in gallium arsenide quantum wells, which are ascribed to many-body interactions.^28^ Third, a small side peak σ at (1.736, 3.312) eV is observed in the absolute diagram, while the real and imaginary parts of the spectra show stronger signals. As the σ peak absorbs approximately twice the |*e*_1_⟩ energy and emits at the |*e*_2_⟩, it suggests that the coherence originates between the |*e*_1_⟩ exciton complexes (i.e., unbound exciton pair 2|*e*_1_⟩ or the bound exciton |*f*_1_⟩) and the single exciton, |*e*_2_⟩. In contrast, we did not observe the coherences between the |*e*_2_⟩ exciton complexes and |*e*_1_⟩, although a slight elongation on top of the β peak in Figure 5e suggests its weak presence. Although the 2Q nonrephasing spectra provided rich information in the multiexciton correlations, certain concerns still remain. One of which is the non-negligible spectral overlap between β and σ in Figures 5d and 5e, leading to ambiguities in deciphering the many-body effects on their presence.

**Figure 5:**
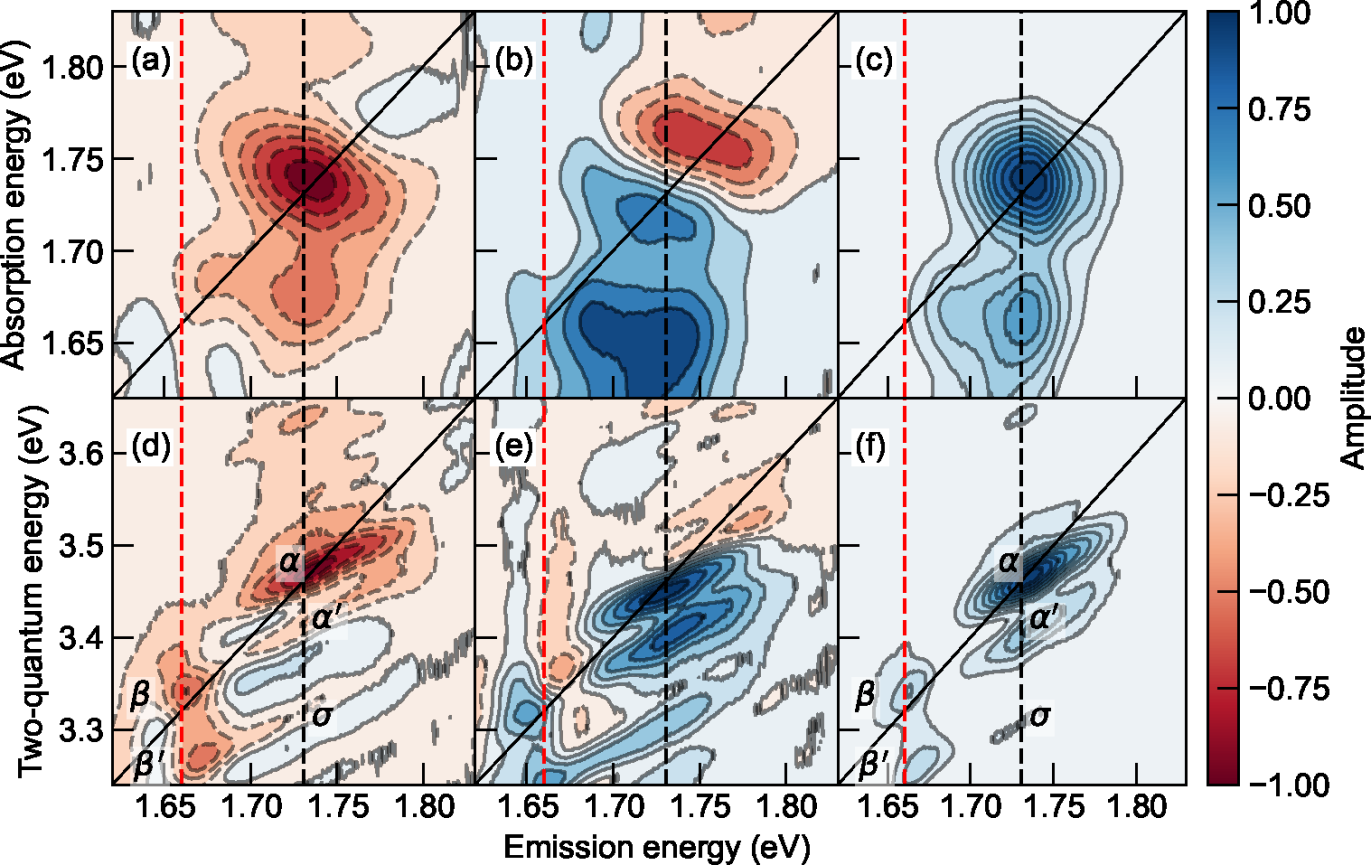
(a) Real, (b) imaginary, and (c) absolute spectrum of the 1Q nonrephasing spectra. (a) Real, (b) imaginary, and (c) absolute spectrum of the 2Q nonrephasing diagram. The black dashed line indicates the peak position at the dominant A_0–1_. The red dashed line locates at the side peak position.

It is worth mentioning that the biexciton states observed here do not originate from the higher-lying excited state. Previous transient absorption measurements on N2200 show that the excited-state absorption lies around 400 meV above the ground-state absorption, which is outside the spectral window here.^44^ In addition, Denti et al. previously conducted Raman and infrared spectroscopy on the doped N2200 system, showing that the polaron formation is strongly localized on the NDI units, in great contrast to other conjugated homopolymers.^45^ Despite the fact that the 2D 1Q measurements performed here look at exciton dynamics at initial population time (*T* = 20 fs), it is not unreasonable to hypothesize that the biexcitons observed in this work might be attributed to interactions between localized excitons on the stacked NDI unit and its neighboring unit. Although we only probed one sample under specific processing conditions, further studies incorporating samples processed under different conditions will be valuable to correlate exciton dynamics with solid-state microstructure which will be essential to understand multiexciton properties in semiconductor polymers. Previously, the short- and long-range aggregation in N2200 have been demonstrated to be tuned by varied molecular weights,^46^ solvent quality,^47^ film annealing,^48^ blending,^49^ etc., which give handles to observe exciton pair and biexciton generations by different preparation processes. Furthermore, to assign the satellite vibrational mode, α, both the energy separation between α and β and Huang–Rhys (HR) factors are needed. Figure 5 suggests that the energy difference between the two vibronic excitons should be larger than 72 meV (580 cm^–1^) as the side peak is limited by the spectral window. The accurate assignment of the vibrational mode other than the dominant ring-stretching mode is still elusive due to unknown HR factors. Nonetheless, if we assume the HR factors of both modes are comparable, the first vibrational mode might land below 1000 cm^–1^, which is in the wavenumber range of the low-energy stretching and torsional modes of the chain backbone because the dominant Raman modes are already around 1500 cm^–1^ in the N2200 thin film. Last but not least, the mixed biexciton state does not seem to contribute significantly in either 1Q or 2Q spectra. As shown by Yang and Mukamel, spectral features from both mixed biexciton states should reside off-diagonally with equal two-quantum energy.^26^ However, the two biexcitons observed in this work do not show coherences from a mixed biexciton state. Only a small interstate coherence peak from pure |*e*_1_⟩ exciton complexes and single exciton |*e*_2_⟩ is observed. Although the electronic transition from |*e*_1_⟩ or |*e*_2_⟩ to |*f*_*m*_⟩ is allowed, the vibrational transition from |*v*_1_⟩ to |*v*_2_, *v*_1_⟩ could be partly forbidden due to the orthogonality of the two normal vibrational modes as indicated in eq 1, where the equality holds true under Born–Oppenheimer (BO) approximation, leading to the weak and even absence of the coherences from the mixed biexciton.
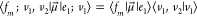
1Previous work by De Sio et al. has demonstrated the presence of conical intersections of multiple potential wells addressed by both symmetric and asymmetric vibrational modes in molecular aggregates.^50^ The BO approximation breaks down close to the conical intersection because the nonadiabatic transition is enabled by vibronic coupling. However, as all measurements performed here are at early population times, the BO approximation should still hold considering that the coherent exciton motion has not yet initiated. Nevertheless, the 2Q spectral features at long population times are of great interest to investigate, as the conical intersection will allow transitions to dark states that are not visible under direct optical excitation.

Finally, we highlight that the pump fluences employed in this work range from 10 to 100 μJ/cm^2^, in which sufficient exciton–exciton annihilation (EEA) is expected in electron push–pull polymers on the picoseconds time scale.^51,52^ Our work shows direct evidence of both correlated exciton pairs and bound biexcitons even at initial population time, which might be precursors for the EEA process in N2200. Dostál et al. directly monitored the change of two-quantum peak intensities for a molecular aggregate in five-wave-mixing experiments with time evolving into the nanosecond range.^20^ By fitting the temporal evolution with the derived theoretical result considering the direct population of biexciton states, they were able to acquire an associated diffusion constant in good consistency with the previous literature.

In addition to the method of direct monitoring through EEI, we suggest that the line shape at initial population time and the diffusion constant might have a deterministic correlation. Moix et al. studied the quantum transport behavior theoretically at short and long times in a one-dimensional J-aggregate chain, when both static disorder and environmental fluctuations exist. Of particular relevance, they treated either analytical solutions for master equations for the exciton dynamics, which correlate the exciton diffusion constants to the Coulombic coupling constant, static disorder, and dephasing rates. In conjugated polymers, the first two parameters can theoretically be acquired by fitting the linear absorption spectra the with the Spano model.^53^ Meanwhile, the dephasing rates could be determined by analyzing the full coherent line shape properly in 2DCS measurements by utilizing the microscopic theory of dephasing.

The microscopic dephasing theory points out that the exciton dynamics generated by the impulsive excitation are not only determined by population decay, which are in turn determined by the radiative and nonradiative rates, but that there is also a contribution to decoherence due to system–bath interactions, e.g., exciton–phonon and exciton–exciton scattering.^27,31^ The combination of both gives rise to the homogeneous line width in the frequency domain, which can be determined by fitting the antidiagonal cut with a Lorentzian function in a purely homogeneously broadened limit. However, in addition to the homogeneous line broadening contributions, the inhomogeneous broadening arising from static disorder (e.g., each molecular segments adopts a slightly different conformation, resulting in different transition energies) will broaden the diagonal line shape, which has an impact on the antidiagonal line width concurrently.^54^ Therefore, alongside the Coulomb coupling constant and the static disorder, the remaining parameter, homogeneous dephasing rate, could be obtained through the line shape analysis; thus, an effective diffusion constant can be determined. The comparison between this and the results determined from traditional ultrafast measurements could lead to new physical insights into the evolution of exciton transport and diffusion behavior.

In conclusion, we perform 1Q and 2Q coherent optical spectroscopic measurements on an electron push–pull conjugated polymer, where clear features originating from two heterogeneous vibronic excitons alongside their exciton complexes are observed. 1Q measurements display spectral features due to the advantageous attractive bound biexcitons, leading to asymmetric cross-peaks. The resultant 2D spectra can be explained qualitatively by tracing the Liouville pathways using a two-exciton model. The 2Q nonrephasing diagram provides further unambiguous evidence of both bound biexcitons and unbound exciton pairs. Specifically, unbound exciton pairs are found to be the dominant feature with a strong attractive biexciton subpeak, the binding energy of which is approximately 70 meV. A weakly repulsive biexciton is also observed from the shoulder of the unbound exciton pairs. The unbound exciton pairs show mixed absorptive and dispersive line shape in contrast to that of the attractive biexciton, indicating the many-body effects in the unbound but correlated exciton pairs.

## Supporting Information Available

Experimental methods including sample preparation, the technique of 2DCS and the XFROG results; additional fluence-dependent 1Q and 2Q measurements are also provided for comparison (PDF)
Transparent Peer Review report available (PDF)
