# Supplementary material for: Unveiling Multiquantum Excitonic Correlations in Push–Pull Polymer Semiconductors
Source: J Phys Chem Lett. 2024 Mar 28;15(14):3705–12. doi: 10.1021/acs.jpclett.4c00065 (PMC11017317; doi:10.1021/acs.jpclett.4c00065)
Supplement: Supplementary file 1 — jz4c00065_si_001.pdf [file jz4c00065_si_001.pdf]

Supporting Information: Unveiling Multi-Quantum Excitonic Correlations in Push-Pull Polymer Semiconductors

Yulong Zheng,[†] Esteban Rojas-Gatjens,[†] Myeongyeon Lee,[‡] Elsa Reichmanis,[‡] and Carlos Silva-Acuña*,[¶,†]

†*School of Chemistry and Biochemistry, Georgia Institute of Technology, 901 Atlantic Drive, Atlanta GA 30332, United States*

‡*Department of Chemical & Biomolecular Engineering, Lehigh University, 111 Research Drive, Bethlehem PA 18015, United States*

¶*Institut Courtois & Département de physique, Université de Montréal, 1375 Avenue Thérèse-Lavoie-Roux, Montréal, Québec H2V 0B3, Canada*

E-mail: carlos.silva@umontreal.ca

Contents

1 Experimental Methods **S3**

1.1 Sample Preparation S3

1.2 Coherent Two-Dimensional Optical Spectroscopy S3

1.3 Pulse Width Characterization S5

2 Additional Experimental Results **S6**

2.1 Fluence-Dependent 1Q Rephasing S6

2.2 Fluence-Dependent 2Q Nonrephasing S7

1 Experimental Methods

1.1 Sample Preparation

N2200 (M_w = 202261 g/mol, PDI = 2.22) was purchased from Ossila Limited. For sample preparation, a solution of 20 g/L in chlorobenzene (anhydrous, Sigma-Aldrich) was prepared by heating at 110°C for 30 minutes, followed by overnight aging at room temperature. The N2200 thin film was fabricated by blade coating on slide glass at 45°C, waiting until the solvent was fully evaporated. A shearing speed of 4mm/s was used to deposit the film.

1.2 Coherent Two-Dimensional Optical Spectroscopy

We employed the experimental setup designed by Turner *et al.*[1] The fundamental beam of 1030 nm was generated using an ultrafast laser system (Pharos Model PH1-20-02-10, Light Conversion) with a repetition rate of 100 kHz. Thereafter, the laser pulse centered around 710 nm was generated through a home-built second harmonic non-collinear optical parametric amplifier. The generated pulse was then directed to the diffractive optical element (DOE). With the zeroth-order beam blocked, the four first-order beams were residing on the four apexes of a square. The superior phase stability and control are achieved through the optical modulation by a reflective two-dimensional liquid crystalline spatial light modulator (SLM).[1] The four beams were compressed individually through the chirp scan[2] and multiphoton intrapulse interference phase scan.[3] Their temporal full widths at half maximum (FWHM) are then characterized through the second-harmonic cross frequency-resolved optical gating (XFROG) .[4] All samples are measured under a high-vacuum vibration-free cryostation at ambient temperature. (Montana Instruments)

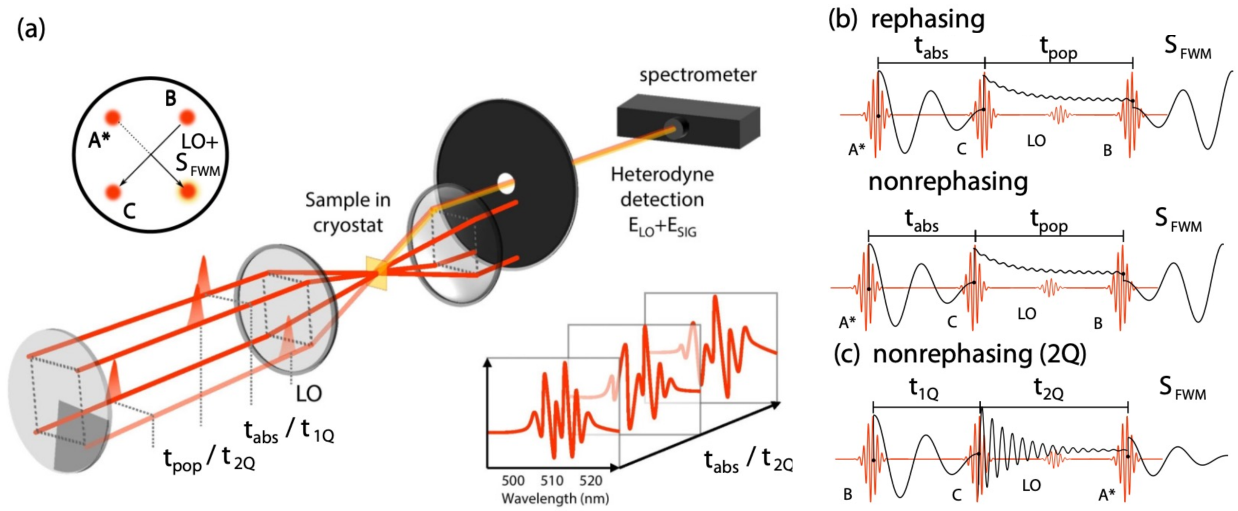

Figure S1: Schematic of the central part of the coherent optical beam recombination technique (COLBERT). (a) The four beams are propagating in the four corners of a square in parallel. Such geometry is called BOXCAR (Box Coherent Anti-Stokes Raman Scattering). The temporal delays, t_{pop} and t_{abs}, and t_{2Q} and t_{1Q} between beams are controlled by the SLM. With such phase relation, the four-wave-mixing signal, S_{FWM} is emitted in the wavevector-conserved direction of the first three beams. The signal is acquired through the heterodyne-detection by interfering with the local oscillator, (LO). (b) The pulse sequences for 1Q rephasing and nonrephasing direction, respectively. (c) Using the same geometry, the 2Q nonrephasing signal can be acquired with alternating pulse sequence. The asterisk indicates the phase-conjugated beam (A*). Figure reproduced from Ref. 5. Copyright 2023 American Physical Society.

1.3 Pulse Width Characterization

The temporal full widths at half maximum for the compressed pulses are fitted by a Gaussian fit, found to be around 17 fs, characterized by XFROG. (Figure S2).

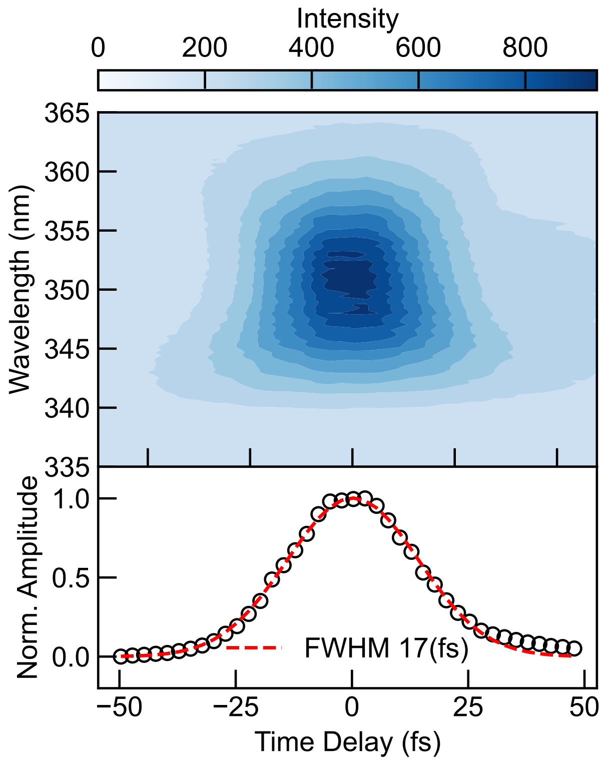

Figure S2: The compressed pulse estimated by a Gaussian fit, characterized by XFROG.

2 Additional Experimental Results

2.1 Fluence-Dependent 1Q Rephasing

The complementary set of fluence-dependent 1Q rephasing diagrams is shown in Figure S3. To more clearly show the dependence of the spectral linewidths, we take the antidiagonal and diagonal cuts of the absolute values of the 1Q rephasing diagrams as displayed in Figure S4. For the antidiagonal cuts, we can readily observe no drastic variance in the spectral linewidths with varying fluences.

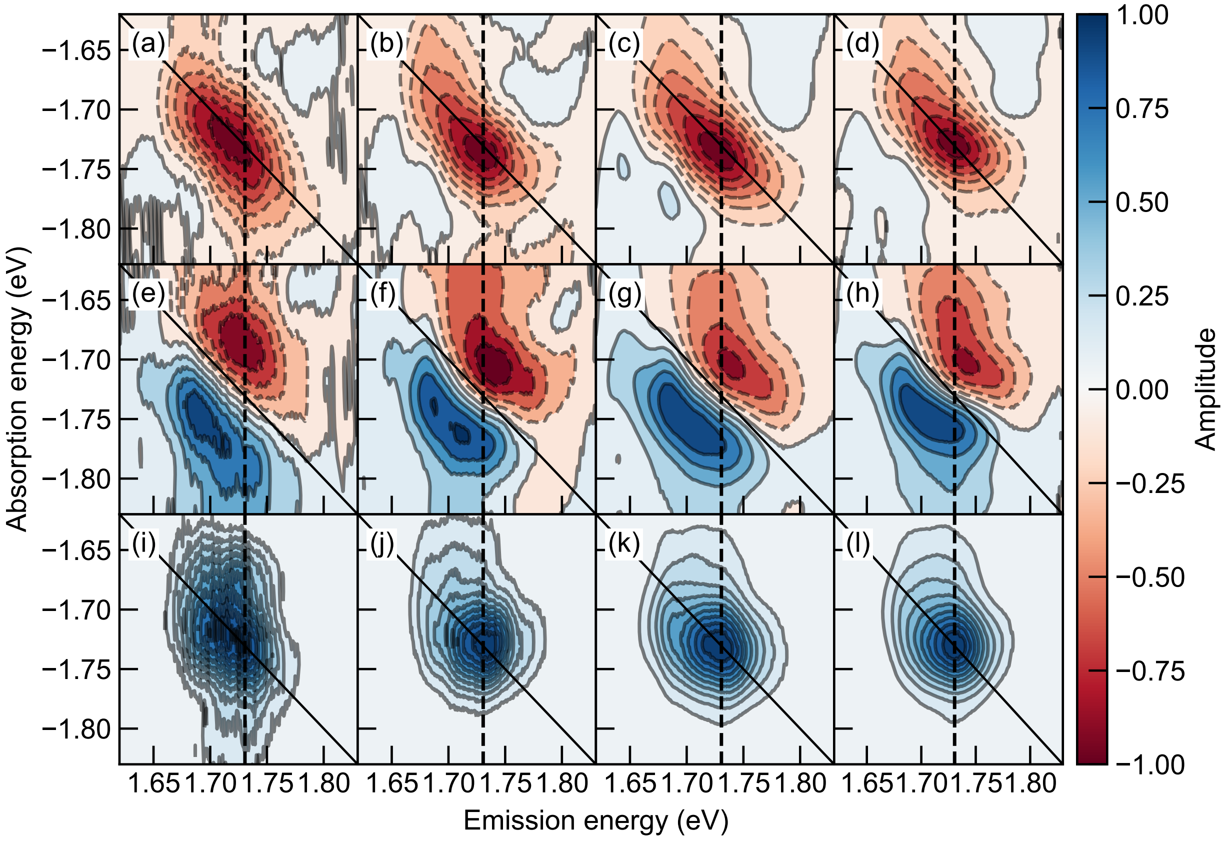

Figure S3: Fluence-dependent 1Q rephasing diagrams. The three rows are real (a-d), imaginary (e-h) and absolute (i-l) values of the 2D spectra. The four columns measured under varying fluences. From left to right are 6.4, 12.7, 51.2 and 121.0μJ/cm^2.

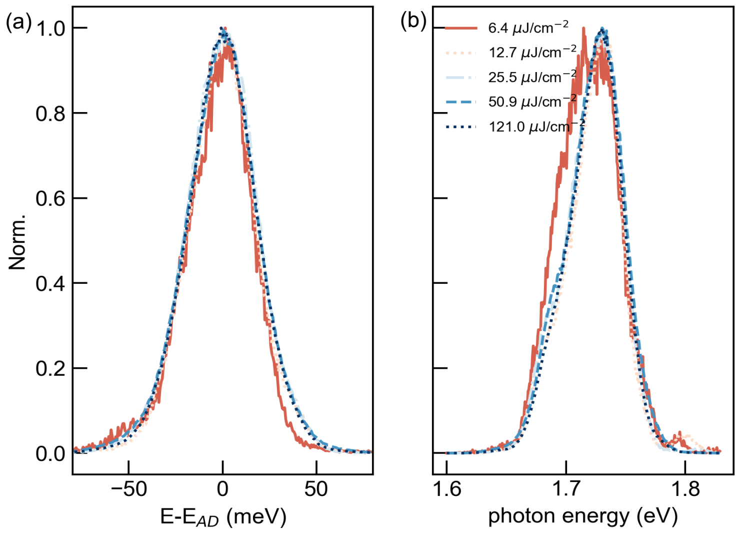

Figure S4: Fluence-dependent antidiagonal (a) and diagonal cuts (b) of the absolute-valued diagrams as shown in Figure S3.

2.2 Fluence-Dependent 2Q Nonrephasing

Here, we show the 2Q measurements performed from low to high fluences (12.7, 25.6, and 51.2 and 121 μJ/cm^2) as shown in Figure S5, the last of which is the same as shown in Figure 5 in the main article, displaying here for a better comparison. All measurements share the dominant features of the two diagonal peaks indicating the two heterogeneous vibronic peaks, each associated with a red-shifted side peak indicating the existence of attractive biexciton states. In addition, certain features are evolving with increasing fluences, including the blue-shifted side peak around (1.66, 3.37)eV and off-diagonal feature at (1.73, 3.30)eV. There could be a possibility of them being an artefact at low fluences, as the modulation efficiency of the spatial light modulator depends on the intensity of the incident light beam.[6] There could also exist possibility of unknown physical processes, which mediate the coupling of the two excitons by fluences. Further quantum dynamics simulations and high-order-wave-mixing experiments are needed to characterize this process.

To show the correlated exciton pairs, attractive and repulsive biexcitons more clearly, we take the vertical cut of the Figure S5d at E_{1Q}=1.736 eV and 1.666 eV. The x-axis is directly adjusted by relation $E_{2Q} - 2E_{1Q}$. Take the dominant vibronic exciton pairs (E_{1Q}=1.736 eV

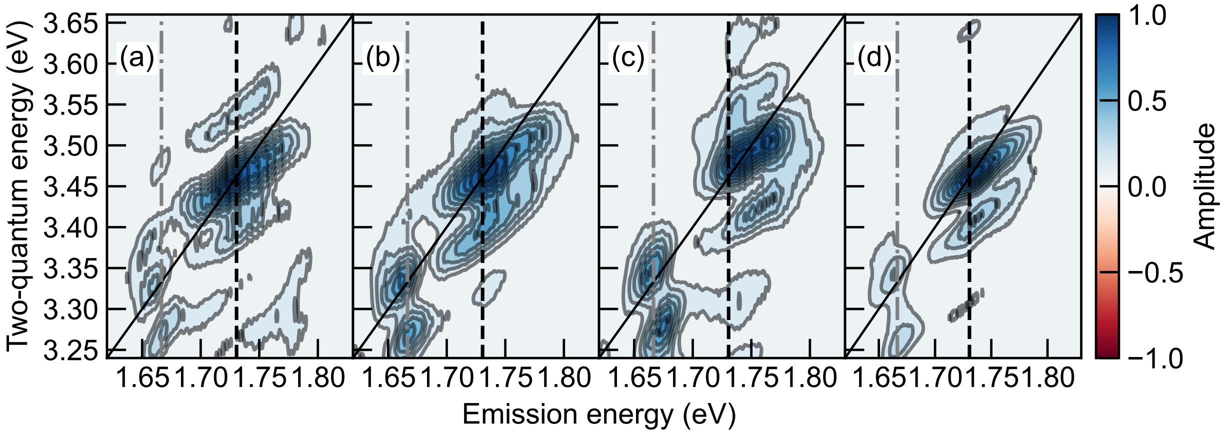

Figure S5: Fluence-dependent 2Q absolute-valued spectra measured under (a) 12.7, (b) 25.6, (c) 51.2 and (d) 121 μJ/cm^2, respectively. The black dashed line indicate the peak position at the dominant A_{0-1}. The grey dashed-dotted line locates at the side peak position.

for example, it can be clearly seen that beside the dominant exciton pairs at ΔE =0 eV, which is the exact double of the E_{1Q}, a red-shifted peak is observed at around -64 meV, indicating an attractive biexciton. In addition, a blue-shifted side peak at 39 meV can also be observed, suggesting the repulsive biexciton state. The side peaks could be observed more clearly in Figure S5a-c, probably with more noise, nevertheless.

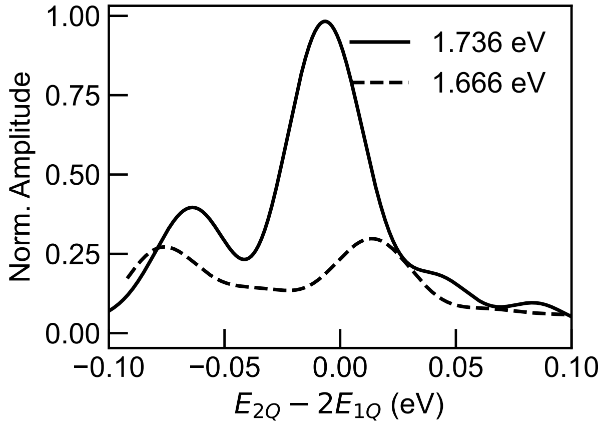

Figure S6: Vertical cuts at E_{1Q}=1.736 eV (solid) and 1.666 eV (dashed) of Figure S5d for the dominant vibronic exciton pairs associated with the both attractive and repulsive biexciton states.

References

(1) Turner, D. B.; Stone, K. W.; Gundogdu, K.; Nelson, K. A. Invited Article: The coherent optical laser beam recombination technique (COLBERT) spectrometer: Coherent multidimensional spectroscopy made easier. *Rev. Sci. Instrum.* **2011**, *82*.

(2) Loriot, V.; Gitzinger, G.; Forget, N. Self-referenced characterization of femtosecond laser pulses by chirp scan. *Opt. Ex.* **2013**, *21*, 24879–24893.

(3) Xu, B.; Gunn, J. M.; Cruz, J. M. D.; Lozovoy, V. V.; Dantus, M. Quantitative investigation of the multiphoton intrapulse interference phase scan method for simultaneous phase measurement and compensation of femtosecond laser pulses. *J. Opt. Soc. Am. B* **2006**, *23*, 750–759.

(4) Amat-Roldán, I.; Cormack, I. G.; Loza-Alvarez, P.; Gualda, E. J.; Artigas, D. Ultrashort pulse characterisation with SHG collinear-FROG. *Opt. Exp.* **2004**, *12*, 1169–1178.

(5) Thouin, F.; Neutzner, S.; Cortecchia, D.; Dragomir, V. A.; Soci, C.; Salim, T.; Lam, Y. M.; Leonelli, R.; Petrozza, A.; Kandada, A. R. S. et al. Stable biexcitons in two-dimensional metal-halide perovskites with strong dynamic lattice disorder. *Phys. Rev. Mater.* **2018**, *2*, 034001.

(6) Vaughan, J. C.; Hornung, T.; Feurer, T.; Nelson, K. A. Diffraction-based femtosecond pulse shaping with a two-dimensional spatial light modulator. *Opt. Lett.* **2005**, *30*, 323–325.
