# Supplementary material for: Unveiling Multiquantum Excitonic Correlations in Push–Pull Polymer Semiconductors
Source: J Phys Chem Lett. 2024 Mar 28;15(14):3705–12. doi: 10.1021/acs.jpclett.4c00065 (PMC11017317; doi:10.1021/acs.jpclett.4c00065)
Supplement: Supplementary file 2 — jz4c00065_si_002.pdf [file jz4c00065_si_002.pdf]

jz-2024-000653.R1

Name: Peer Review Information for "Unveiling Multi-Quantum Excitonic Correlations in Push-Pull Polymer Semiconductors"

First Round of Reviewer Comments Reviewer: 1

Comments to the Author

Journal: The Journal of Physical Chemistry Letters

Manuscript ID: jz-2024-000653
Original Submission Date: 08-Jan-2024
Title : "Unveiling Multi-Quantum Excitonic Correlations in Push-Pull Polymer Semiconductors"
Author(s): Zheng, Yulong ; Rojas-Gatjens, Esteban; Lee, Myeongyeon; Reichmanis, Elsa; Silva-Acuña, Carlos

The authors present an argument for their interpretation of 2D spectral features of N2200, in which they find that unbound, yet correlated, exciton pairs contribute more prominently to the spectra than biexcitons. The biexciton populations feature a very weak repulsive contribution and a strong attractive contribution. The authors Distinguishing between these populations' contributions to the 2D spectra by using both 1Q and 2Q experiments adds a more complete concept for communication in the Journal of Physical Chemistry Letters. A large portion of the novelty of this letter resides in the material system as well, using an inorganic semiconductor to describe many-body correlations in systems featuring Frenkel excitons.

Detailed remarks and technical suggestions:

1. The work could be greatly improved by further discussion of the relationship between aggregation in the disordered film and the nature of the exciton pairs/biexciton contribution to the 2D spectra. In the introduction, the authors present an argument that the varied morphology of organic semiconductors contributes to the nature of the exciton pairs present at high fluences. While they cite literature about aggregate types and their impact on delocalization of the exciton wavefunction and exciton-exciton interaction, I find that the original work presented in the results and discussion section does not sufficiently delve into these ideas.
    a. The most scientifically rigorous approach, that would certainly uphold the standards of Journal of Physical Chemistry Letters, would be to acquire a new

N2200 sample prepared differently (for example bypassing the thermal annealing step, processing from a different solvent, varying the average polymer chain size, using an additive that supports or interrupts aggregation, etc.), and repeat the 1Q and 2Q measurements to compare the impact of aggregation on the results.
   b. If the authors have justified practical limitations to acquiring a sample or repeating the experiments, a coherent discussion citing morpohological and spectral results of N2200 already present in literature with clear links to the data presented in this manuscript could suffice.
2. Though the authors clearly communicate that the line shape did not change significantly throughout the range of fluence conditions, the following discussion on fluence-dependence and exciton-exciton interactions discussed in the paragraph that begins "Finally, we highlight that the pump fluences…" is difficult to follow. I suggest breaking this into multiple paragraphs with more explicit links to the original data presented in the manuscript.
3. The caption for Figure 2 describes both (b)-(d) and (f)-(h) as rephasing when (f)-(h) should be non-rephasing.

Reviewer: 2

Comments to the Author

This paper presents a 2DCS study of Frenkel-exciton complexes focused on the direct measurement of the binding energy using 1Q and 2Q spectra with evidence shown for negative as well as positive exciton-exciton coupling.

The paper is written exceptionally clearly, the arguments that are given are convincing, this reviewer (who is more of an informed and curious outsider rather than an expert) sees no evidence or reasons to doubt the interpretation of data, the conclusions, or the exciting outlook. The discussion thoughtfully provides explanations for why expected features appear too weak/strong and removes doubts, as well as appropriately discusses remaining challenges. I recommend publication more or less as is with minor adjustments intended to help reach a broader audience.

To this end, I have a couple of questions and a couples of suspected typos to bring to your attention.

Q1: I did not quite understand the argument that the transition from |1, 1> to |0,2> or |2,0> is forbidden because vibrational modes are orthogonal, it would be terrific if an equation supporting this intuition was included.

Q2: Fig 5d-f: It is somewhat counterintuitive or at least it strikes a non-expert eye as self-contradictory to infer that the coupling is both negative (binding energies are around -70meV) and positive (effective coupling ~30meV). If a Frenkel exciton Hamiltonian was being parameterized using this spectrum – what magnitude of the coupling would be used? If I misunderstood the text,

non-expert (or pre-expert) readers would benefit from that part getting a slight upgrade, perhaps including precise definition of what is meant by the binding energies, effective couplings etc close to this discussion.

Typos:

1. Repeated ‘and’ in introduction: In this work, we show direct evidence of two distinct excitons dressed by different vibrational modes, each with its own vibronic progression, and Frenkel biexcitons and correlated exciton pairs in one-quantum (1Q) and two-quantum (2Q) two-dimensional coherent spectra (2DCS).

2. Caption to Figure 3:’ … highlight negative features indicating…’: the features mentioned here are blue and therefore positive in this version of the figure.

Author's Response to Peer Review Comments:

Dear Editor,

Many thanks for transmitting the referee reports, which we have responded to fully in the attached document. We hope that the manuscript is now acceptable for publication in JPCL, and we look forward to hearing from you in due course.

Best regards,

Carlos Silva

Reviewer(s)' Comments to Author:

Reviewer: 1

Recommendation: This paper is publishable subject to minor revisions noted. Further review is not needed.

Comments:

The authors present an argument for their interpretation of 2D spectral features of N2200, in which they find that unbound, yet correlated, exciton pairs contribute more prominently to the spectra than biexcitons. The biexciton populations feature a very weak repulsive contribution and a strong attractive contribution. The authors distinguishing between these populations' contributions to the 2D spectra by using both 1Q and 2Q experiments adds a more complete concept for communication in the Journal of Physical Chemistry Letters. A large portion of the novelty of this letter resides in the material system as well, using an inorganic semiconductor to describe many-body correlations in systems featuring Frenkel excitons.

We appreciate the reviewer's positive feedback on this manuscript. We address each comment as follows color-coded in red for the reviewer and for the editor's convenience to track our edits.

Responses to reviewers' comments

1. The work could be greatly improved by further discussion of the relationship between aggregation in the disordered film and the nature of the exciton pairs/biexciton contribution to the 2D spectra. In the introduction, the authors present an argument that the varied morphology of organic semiconductors contributes to the nature of the exciton pairs present at high fluences. While they cite literature about aggregate types and their impact on delocalization of the exciton wavefunction and exciton-exciton interaction, I find that the original work presented in the results and discussion section does not sufficiently delve into these ideas.

   We thank the reviewer's comment on questioning the relationship between the polymer

morphologies and exciton pairs generation. Indeed the varied polymer microsctructures might induce different photophysical aggregates (H or J type). However, we want to emphasize that the length scale that are related to the exciton pairs and/or biexcitons is on the order of a few monomers, which is directly correlated with the exciton interactions and exciton wavefunction delocalization. This should validate our reasoning and focusing on photophysical aggregate types instead polymer morphological influences on the exciton behavior. That being said, we do acknowledge the fact that there is indirect impact of varying polymer phases and morphologies on photophysical behavior, thus exciton dynamics. We now acknowledged the importance between microstructures and the exciton dynamics, and referenced the important work of varying processing conditions from the previous literature. The detalied modifications are shown below.

a. The most scientifically rigorous approach, that would certainly uphold the standards of Journal of Physical Chemistry Letters, would be to acquire a new N2200 sample prepared differently (for example bypassing the thermal annealing step, processing from a different solvent, varying the average polymer chain size, using an additive that supports or interrupts aggregation, etc.), and repeat the 1Q and 2Q measurements to compare the impact of aggregation on the results.

We thank the reviewer's comment on relating the morphological changes of N2200 to the exciton pairs. Alongside some of the reasons we mentioned above, we do agree with the reviewer that a systematic study of the exciton pairs with N2200 thin films with varied morphology, molecular weights or chain lengths might be of great interest and significance to not only spectroscopy but also conjugated polymer community. However, the main focus of this work is to understand the 2D coherent spectral lineshapes due to exciton pairs in both 1Q and 2Q measurements, and conducting a, extensive new set of experiments might would a significant amount of effort and time that would not significantly add to the objectives of this work. Nevertheless, we do agree that a follow-up effort to correlate these interactions with solid-state microstructure such

systematically is important. On page 13 line 23, we add the acknowledgement in the fundamental interest in the dependence of solid-state microstructure:

"...and its neighboring unit. Although we only probed one sample under specific processing conditions, further studies incorporating samples processed under different conditions will be valuable to correlate exciton dynamics with solid-state microstructure which will be essential to understand multi-exciton properties in semiconductor polymers."

b. If the authors have justified practical limitations to acquiring a sample or repeating the experiments, a coherent discussion citing morphological and spectral results of N2200 already present in literature with clear links to the data presented in this manuscript could suffice.

We appreciate the reviewer's comment and considerations. We add extra discussion regarding the structure-property relationship in N2200 as follows.

On page 13 line 23 in the original manuscript:

"Previously, the short- and long-range aggregation in N2200 have been demonstrated to be tuned by varied molecular weights[45], solvent quality[46], film annealing[47], blending[48] and etc, which give handles to observe exciton pair and biexciton generations by different preparation processes."

2. Though the authors clearly communicate that the line shape did not change significantly throughout the range of fluence conditions, the following discussion on fluence-dependence and exciton-exciton interactions discussed in the paragraph that begins "Finally, we highlight that the pump fluences…" is difficult to follow. I suggest breaking this into multiple paragraphs with more explicit links to the original data presented in the manuscript.

We thank the reviewer's comment. We want to direct this last paragraph for studies of interest in analyzing the line shape, which will reflect the impact of exciton exciton annihilation. To make to the last second paragraph more accessible to the broader

readership, we separated the paragraph into multiple ones and made the following adjustments:

On page 14 line 23, we add:

"...electron push-pull polymers on picoseconds time scale. Our work shows direct evidence of both correlated exciton pairs and bounded biexcitons even at initial population time, which might be precursors for EEA process in N2200."

On Page 15 line 19:

"~~By accurately acquiring the homogeneous dephasing rates, combined with~~ Therefore, alongside the Coulomb coupling constant~~s~~ and the static disorder, the remaining parameter, homogeneous dephasing rate, could be obtained through the lineshape analysis, thus, an effective diffusion constant can be determined."

3. The caption for Figure 2 describes both (b)-(d) and (f)-(h) as rephasing when (f)-(h) should be non-rephasing.

   We thank the reviewer's catch on the caption. Now it is fixed.

Comments:

Additional Questions: Urgency: Moderate

Significance: High

Novelty: Top 10%

Scholarly Presentation: High

Reviewer: 2

This paper presents a 2DCS study of Frenkel-exciton complexes focused on the direct measurement of the binding energy using 1Q and 2Q spectra with evidence shown for negative as well as positive exciton-exciton coupling.

The paper is written exceptionally clearly, the arguments that are given are convincing, this reviewer (who is more of an informed and curious outsider rather than an expert) sees no evidence or reasons to doubt the interpretation of data, the conclusions, or the exciting outlook. The discussion thoughtfully provides explanations for why expected features appear too weak/strong and removes doubts, as well as appropriately discusses remaining challenges. I recommend publication more or less as is with minor adjustments intended to help reach a broader audience.

To this end, I have a couple of questions and a couples of suspected typos to bring to your attention.

We appreciate the reviewer's positive and encouraging feedback. We want to further address the comment individually down below. We made modifications color-coded in blue for the reviewer and editor's convenience on tracking our edits.

1. I did not quite understand the argument that the transition from $|1,\ 1\rangle$ to $|0{,}2\rangle$ or $|2{,}0\rangle$ is forbidden because vibrational modes are orthogonal, it would be terrific if an equation supporting this intuition was included.

   The statement, the transition from $|1,\ 1\rangle$ to $|0{,}2\rangle$ or $|2{,}0\rangle$ is forbidden' is true under the one-photon transition condition, meaning that only one-photon-led process is achieved for each light beam-matter interaction. For example, $|1,\ 1\rangle$ to $|0{,}2\rangle$ would need one photon to deexcite from the first vibronic exciton to ground state and a sequential photon to excite the second vibronic exciton to the biexciton state. Such process needs two beam to achieve.

   The second part addresses the magnitude of transition dipole moments where the orthogonal vibrational modes come from the equation below:

$$\langle f_m; v_1, v_2|\vec{\mu}|e_1; v_1\rangle = \langle f_m|\vec{\mu}|e_1\rangle\langle v_1, v_2|v_1\rangle \tag{1}$$

The equation holds true under Born-Oppenheimer approximation and the first term on the right hand side is nonzero. But the second term could be small or zero if the orthogonal vibrational mode, relative to the singly-excited vibronic state, is dominant. The exact value is dependent on the accurate description of the vibrational wavefunction.

On page 13 line 51, we modify:

~~We rationalize it as the two vibrational modes should be orthogonal under the Born-Oppenheimer approximation (BO), therefore the transition from $|e_1\rangle$ or $|e_2\rangle$ to $|f_m\rangle$ is forbidden.~~ "Although the electronic transition from $|e_1\rangle$ or $|e_2\rangle$ to $|f_m\rangle$ is allowed, the vibrational transition from $|v_1\rangle$ to $|v_2, v_1\rangle$ could be partly forbidden due to the orthogonality of the two normal vibrational modes as indicated in Equation 2, where the equality holds true under Born-Oppenheimer (BO) approximation, leading to the weak and even no appearance of the coherences from the mixed biexciton.

$$\langle f_m; v_1, v_2|\vec{\mu}|e_1; v_1\rangle = \langle f_m|\vec{\mu}|e_1\rangle\langle v_1, v_2|v_1\rangle \tag{2}$$

2. Fig 5d-f: It is somewhat counterintuitive or at least it strikes a non-expert eye as self-contradictory to infer that the coupling is both negative (binding energies are around -70meV) and positive (effective coupling 30meV). If a Frenkel exciton Hamiltonian was being parameterized using this spectrum – what magnitude of the coupling would be used? If I misunderstood the text, non-expert (or pre-expert) readers would benefit from that part getting a slight upgrade, perhaps including precise definition of what is meant by the binding energies, effective couplings etc close to this discussion.

We appreciate the reviewer's comment on the both negative and positive features

observed in this system. First, we want to apologize for the wording here, both energies should be give as binding energies rather coupling constant rigorously. We now took a vertical spectral cut at E_{1Q}=1.73 eV as shown in the SI Figure S6. Beside the dominant on-diagonal peak, we can see a clear red-shifted peak which is attributed to the attractive biexciton state, alongside a weaker blue-shifted shoulder which is attributed to the Pauli exclusion of exciton-exciton scattering. Experimentally, the binding energies can be found as $E_{2Q} - 2E_{1Q}$ from the spectral shifts. Furthermore, we want to highlight that the system should be understood as a Hubbard-like model for the dynamic exciton-exciton interactions [Agranovich, V.M., *et al.*, 2000, J. Lumin; Knoester&Agranovich, 2003, Thin Films & Nanostruct., Vol. 31]:

$$\widehat{H}_{\text{ex}} = \sum_{n}(\hbar\omega_0 + D_n)b_n^{\dagger}b_n + \sum_{n,m} J_{nm}b_n^{\dagger}b_m + \frac{1}{2}\sum_{n,m} U_{nm}b_n^{\dagger}b_m^{\dagger}b_n b_m. \quad (3)$$

Therefore, the attractive energy should be parameterized as the dynamical exciton-exciton interaction in the last term. If ignoring the dynamic EEI term, the commutation term for bosons will inevitably lead to doubly excited molecules which are prohibited as mentioned above. This will lead to an effective two-exciton state which looks exactly like an actual biexciton state. Previously, such model has already been well-established and employed in organic materials.[Fidder, H *et al.*, 1993, J. Chem. Phys.]

To show the red- and blue-shifted side peaks more clearly, We now add an additional peak with vertical cut of the dominant vibronic peak in SI Figure S6.

On page 12 line 17, we now determine the values of binding energies more accurately based on the spectral cut, we add:

"Therefore, the binding energies, experimentally determined as $(E_{2Q} - 2E_{1Q})$, for $|f_1\rangle$ and $|f_2\rangle$ are estimated to be -76 and ~~-70 meV~~ -64 meV as shown in Figure S6, respectively, where the negative sign indicates their attractive nature. The exciton binding

energies are comparable since the two vibronic excitons have the same electronic origins, while the slight difference."

On page 12 line 25:

"Therefore, the ~~effective coupling strength~~repulsive binding energy can be estimated to be around ~~30~~39 meV."

3. Typos:

a. Repeated 'and' in introduction: In this work, we show direct evidence of two distinct excitons dressed by different vibrational modes, each with its own vibronic progression, and Frenkel biexcitons and correlated exciton pairs in one-quantum (1Q) and two-quantum (2Q) two-dimensional coherent spectra (2DCS).

On page 3 line 21 we paraphrased as follows:

"progression~~, and~~. Furthermore, we demonstrate the presence of Frenkel biexcitons and correlated exciton pairs revealed in one-quantum (1Q) and two-quantum (2Q) two-dimensional coherent spectra (2DCS)."

b. Caption to Figure 3:' ... highlight negative features indicating...': the features mentioned here are blue and therefore positive in this version of the figure.

We thank the reviewer's notice and now it is changed to positive.

Additional Questions: Urgency: High

Significance: High

Novelty: High

Scholarly Presentation: High

Is the paper likely to interest a substantial number of physical chemists, not just specialists working in the authors' area of research?: Yes

Per the editor's comments:

1. Title: In both the main manuscript file and the Supporting Information, set the title in title case, with the first letter of each principal word capitalized.

   Now the first letters are made capitalized.

2. Abstract: Shorten the abstract to 150 words or fewer.

   We now shortened the abstract with the modifications color-coded in violet.

3. Title and Author Lists: Title, author names, and affiliations must match in three places: (1) manuscript file, (2) supporting information, and (3) ACS Paragon Plus. Affiliations don't match.

   We now matched the affiliations between the manuscript and ACS Paragon Plus.

4. References: In both the main file and the supporting information, fix the style of all references to use JPCL formatting (check all references carefully). ***JPC Letters reference formatting requires that journal references should contain: () around numbers; author names; article title (titles entirely in title case or entirely in lower case); abbreviated journal title (italicized); year (bolded); volume (italicized); and pages (first-last). Book references should contain author names; book title (in the same pattern); publisher; city; and year. Websites must include date of access.

   The format is now adjusted to conform to JPCL's requirement.

5. Supporting Information: Please number SI pages in the following format: "S1, S2..."

   We adjusted the number of SI pages in the format as required.

6. Graphics: One or more of your figures and tables includes a reference citation. Please confirm that this pertains only to data and not the figure itself. If it pertains to the use of a published image, permissions must be secured for any graphics NOT originally published by ACS or for Open Access content which permits reuse with credit only. Permission is needed if you are using another publisher's or copyright

owner's figures/tables verbatim, adapting/modifying them, or using them in part. If the images are from an Open Access publisher that does not require permission for reuse, please confirm.

For the one that is reproduced in the main manuscript, it only pertains to the data quality. For the one in the SI that shows the schematic of COLBERT, we already required the permission from the American Physical Society.
